# Supplementary material for: Auditory or Audiovisual Stimulation Ameliorates Cognitive Impairment and Neuropathology in ApoE4 Knock-In Mice
Source: Int J Mol Sci. 2023 Jan 4;24(2):938. doi: 10.3390/ijms24020938 (PMC9863367; doi:10.3390/ijms24020938)

Table S1. Results of the Morris water maze test in the ApoE4-KI mice after auditory or audiovisual stimulation.

	N	A	AV	*P*-value between times (F value)	*P*-value between groups (F value)	*P*-value interactions (F value)
Distance moved (cm)						
Day 0	262.86±157.76	304.91±152.02	180.72±99.12	ns (0.1928)	ns (0.2353)	ns (0.4829)
Day 7	244.48±65.76	176.88±79.29	191.75±77.73			
Day 14	236.21±80.2	170.86±46.24	234.86±132.45			
Velocity (cm/s)						
Day 0	15.82±2.43	22.16±7.55	15.6±4.03	ns (2.177)	ns (0.04905)	ns (0.2064)
Day 7	13.82±2.85	21.92±8.07	15.13±2.39			
Day 14	13.68±1.22	20.93±6.15	19.38±5.70			
Moving percent (%)						
Day 0	97.72±0.2	90.56±8.94	85.16±12.86	ns (0.6383)	ns (1.581)	ns (0.4384)
Day 7	96.96±0.98	96.61±1.17	96.99±1.00			
Day 14	98.52±0.38	98.08±1.24	96.77±2.11			
Not moving percent (%)						
Day 0	0.59±0.35	8.79±8.98	14.22±12.88	ns (0.8195)	ns (1.439)	ns (0.4760)
Day 7	2.00±1.26	2.72±1.14	2.51±1.00			
Day 14	0.44±0.44	1.40±1.26	2.70±2.18			
Percent time in quadrant (%)						
Day 0	42.27±5.74	38.94±7.67	49.69±6.52	ns (1.900)	ns (3.568)	ns (2.611)
Day 7	29.77±4.88	41.84±7.28	32.87±3.52			
Day 14	32.75±5.89	41.31±8.04	41.41±7.07			
Absolute escape latency (s)						
Day 0	18.4±10.86	19.48±11.61	12.78±5.95	ns (0.7339)	ns (0.3360)	ns (0.4332)
Day 7	21.43±7.08	11.07±7.16	13.98±5.40			
Day 14	16.80±4.63	9.40±2.07	13.10±6.80			
Relative escape latency (ratio)						
Day 0	1±0.59	1±0.6	1±0.47	ns (1.165)	ns (0.2335)	ns (0.3123)
Day 7	1.16±0.38	0.57±0.37	1.09±0.42			
Day 14	0.91±0.25	0.48±0.11	1.03±0.53			

Values are expressed as mean ± SEM. Statistical analysis was performed using a two-way ANOVA followed by Bonferroni's multiple comparisons test. N, no stimulation; A, auditory stimulation; AV, audiovisual stimulation; NS, nonsignificant.

Figure S1. Results of the Morris water maze test in the ApoE4-KI mice after auditory and audiovisual stimulation.

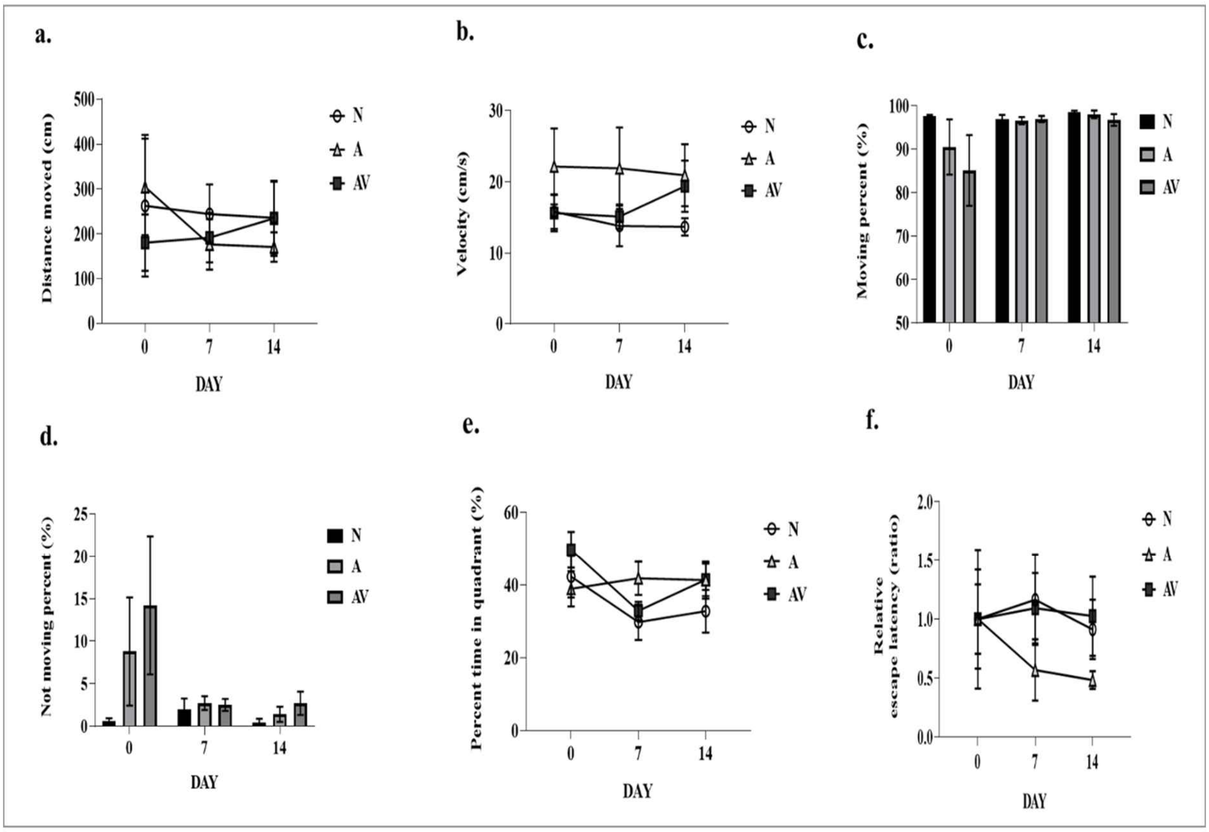

a. Distance moved; b. Velocity; c. Moving percent; d. Not moving percent; e. Percent time in quadrant; f. Relative escape latency. N, no stimulation; A, auditory stimulation; AV, audiovisual stimulation.

Figure S2: The original images for western blot analysis of apoptosis-related proteins in ApoE4-KI mice after auditory and audiovisual stimulation.

a. Bax

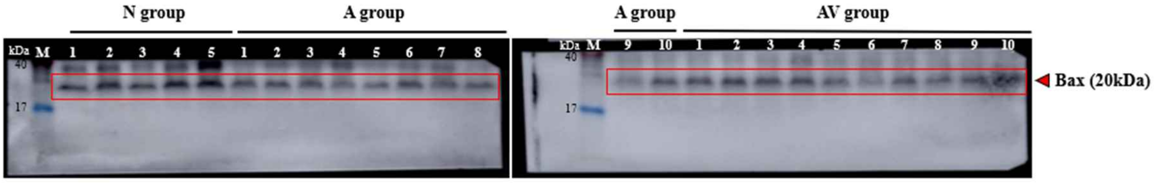

b. Bcl-2

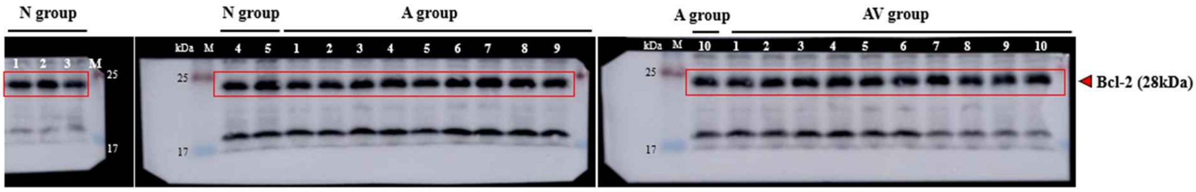

c. Caspase 3

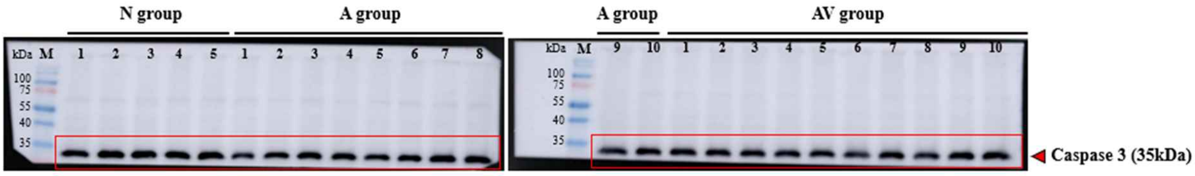

d. β-actin

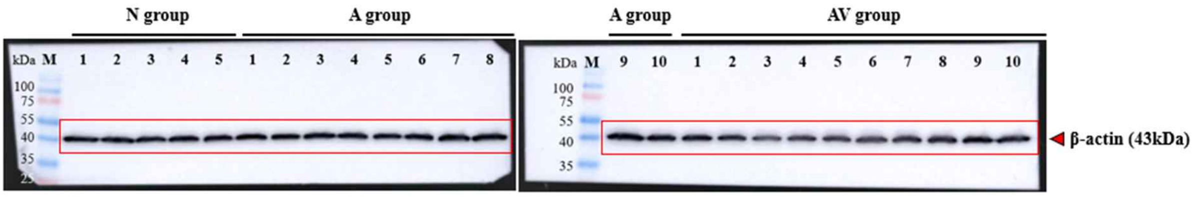

Supplement: Supplementary file 1 [file ijms-24-00938-s001.zip › ijms-2112594-supplementary.pdf]
